# A case report of anorexia nervosa in a 23‐year‐old Ethiopian woman

**DOI:** 10.1002/ccr3.8150

**Published:** 2023-11-20

**Authors:** Selamawit Alemayehu Tessema, Surafel Worku Megersa, Meskerem Abebe, Hailegiorgis Getnet Ayalew, Melat Tigabu

**Affiliations:** ^1^ Psychiatry department St. Paul Hospital Millennium Medical College Addis Ababa Ethiopia

**Keywords:** anorexia nervosa, eating disorders, Ethiopia, management of anorexia nervosa

## Abstract

**Key Clinical Messages:**

Limited studies on AN in Africa, including Ethiopia. Internet and media have changed body image portrayal in developing countries. A need for a multidisciplinary approach to treatment, training on positive coaching styles, and future research.

**Abstract:**

The Global Burden of Disease had estimated anorexia nervosa (AN) or bulimia nervosa to be 13.6 million people. The lifetime prevalence of AN ranges from 2.4 to 4.3 percent. During their lifetime, up to 4% of females and up to 0.3% of males suffer from anorexia nervosa. Studies assessing AN in Africa, including Ethiopia, are limited. This case report describes a 23‐year‐old female patient who presented with anorectic symptoms and signs in Ethiopia. This case report describes a 23‐year‐old female patient who participated in sports activities. She had a low body weight based on a BMI of 13.15 kg/m^2^ and lost around 10 kg within the past 6 months. She feared gaining weight or becoming fat, thus restricting food intake. The findings on psychiatric evaluation encompassing detailed history and mental state examination suggested the diagnosis of Extreme anorexia nervosa, restricting type; adult malnutrition; major depressive disorder (MDD) (in remission); low risk of aggression; low risk of suicide; severe functional impairment. The general management principles implemented in this patient included assessment of medical complications, weight restoration, psychological intervention, medication for comorbid depression, and long‐term psychological and biological treatment follow‐up to avoid relapse. The presented case of a 23‐year‐old Ethiopian female patient who is athletic shows weight‐controlled sports activities and the manifestation of anorexia nervosa. Easy access to the Internet and media has changed body image portrayal in developing countries including Ethiopia. There is a need for a multidisciplinary approach involving psychiatrists, psychologists, internists, and nutritionists for the management of AN. The early screening and management of medical complications are crucial. There is a need for close monitoring of vital signs, restriction of caffeine, excess fluid, and salt, and limiting excessive exercise. Furthermore, to assess micronutrient deficiencies, vitamin supplements should be prescribed in the form of multivitamin and thiamine preparations. The need for future training about positive coaching styles for coaches is mandatory to reduce the future impacts on young athletes. There is a need for future research on eating disorders in developing countries such as Ethiopia.

## 1 INTRODUCTION

Anorexia nervosa is one of the eating disorders that result in restricting food intake because of fear of gaining weight or becoming obese resulting in significantly low body weight with BMI below 17.5 for adults that is present for 3 months.^1^, ^2^, ^3^, ^4^ Furthermore, may involve other behaviors that will affect weight like excessive exercise and purging.^1^, ^2^

According to the Global Burden of Disease (GBD), in 2019 an estimated 13.6 million people had anorexia nervosa or bulimia nervosa which is equivalent to 176.2 per 100,000 people.^5^ The GBD showed that eating disorders including solely anorexia nervosa and bulimia nervosa were ranked the 110th cause of disability‐adjusted life years (DALY).^5^, ^6^ Anorexia Nervosa can be a severe and disabling condition with negative impacts on individuals and family members.^6^

The lifetime prevalence of anorexia nervosa ranges from existing epidemiological studies ranges between 2.4% and 4.3%.^4^, ^7^ During their lifetime, up to 4% of females and up to 0.3% of males suffer from anorexia nervosa.^7^ The peak age of onset is during adolescence.^8^ The onset of age in young women ranges peaks between ages 13 and 14 years and 18 and 20 years.^9^ The new case detection rate (incidence rate) of anorexia nervosa has been stable in most studies in the last 10 years. New incidence among young below 15 years has increased over the years. Several studies suggest that this might be due to early screening and diagnosis.^7^ Individuals with anorexia nervosa might be unaware of the medical complication and the significant weight loss when others may be concerned about it. The increased medical complications and suicide can increase the mortality due to anorexia nervosa to as high as five or more times increased.^1^, ^7^ The mortality rate for eating disorders is among the highest of any psychiatric illness.^10^, ^11^

Although anorexia nervosa is most commonly seen in the Western race and culture, it can also occur in males and females of all ages, races, and cultural backgrounds.^2^ Several studies suggest there is gender preponderance elicited with females than males.^2^, ^7^ Evidence suggests the age of onset in males varies; males tend to present at late age compared with females.^2^ This might be due to poor health‐seeking behavior in males and underdiagnosis. This can give the assumptions of stigma toward psychiatric disorders, limited community‐based studies, and stigma of suffering from a “female‐specific” disorder can contribute to the underdiagnosis.^7^

Cultural factors within a society influence beauty ideals. These factors can be mass media, peer interaction, and parental influence.^12^ The body size ideals might vary from country to country. In Africa, the body ideals might vary on the country of residence and their sociocultural status. Those residing on low incomes with isolated from Western culture still might have an ancestral idea of beauty, preferring a shapely body.^13^ Evidence suggests that currently there is no wide difference in body size ideals in Western and non‐Western cultures. This is also the case in most urban settings of Africa including Ethiopia where there is modernization and westernization that promote thin body ideals.^12^ According to the review done by van Hoeken and colleagues on eating disorders in Africa, the estimated coverage of studies on eating disorders in eastern sub‐Saharan Africa is 0.1%.^14^ In this study, the pooled point prevalence rate of women in Africa was 0.87% and 4.45% for bulimia nervosa and eating disorder not otherwise specified, respectively.^14^ There were no cases of DSM‐IV anorexia nervosa reported among a sample of 1476 women in four epidemiological studies in Africa. These show that little or no cases of anorexia nervosa diagnosis were reported in African epidemiological studies. There is an overall misconception that anorexia nervosa is rare in Africa despite this belief there is an emerging reports of cases from African descent. Several cases were reported in Nigeria,^15^, ^16^ South Africa^17^ Etc. In the Ethiopian context, there is a case report done on a 17‐year‐old girl with the diagnosis of bulimia nervosa.^18^ Apart from the above case report up to the knowledge of authors no published report of anorexia nervosa in the Ethiopian context.

Risk factors that are associated with anorexia nervosa include genetics, body dissatisfaction, low self‐esteem, perfectionist personality, peer influence, social media body image influence, history of bullying, negative comments about body weight, and others.^19^ Studies suggest that a negative coaching style that only focused on athletes' weight gain, the impact on team score, competitiveness, and performance can have an impact on the athletes' body image, weight preoccupation, and dieting.^20^ The sports environment can make athletes vulnerable to risk factors of anorexia nervosa and other eating disorders. The study identifies sport‐specific risk factors like dieting and experienced pressure to lose weight, personality traits, early age at the start of training, impression motivation, and body weight preoccupied coaching style.^19^, ^20^

There are limited community‐based studies worldwide and all available published reports agree on the rare incidence of anorexia nervosa.^2^ Most of the existing epidemiological studies on anorexia were conducted in Western countries.^7^ In Africa including Ethiopia studies assessing anorexia nervosa are scarce.^7^, ^14^ This case reflects a 23‐year‐old female patient who presented with anorectic symptoms and signs in Ethiopia.

## 2 CASE PRESENTATION

This is a 23‐year‐old female patient who is currently visiting our hospital (SPHMMC) for the first time. She is single and holds a degree in public health. She lives with her mother and is the only child in the family. She presented with an experience of easy fatigability for the past 6 months, loss of appetite, and gradual weight loss for the past 9 years.

She has been dieting and exercising since 9 years back by that time she was a grade 7 student. She started a taekwondo class around her village with her friends. She worksouts 4 days per week for about 3–4 h daily. She states that the workout was intense and exhausting despite her enjoying the sport. She was more competitive and was involved in different games by representing the taekwondo school. She claims that their coach was a strict person who recommended different diets to eat and not to eat. Furthermore, he also comments on their weight saying “How will you jump with such weight” if someone gains weight. During days when there is an upcoming game, he will measure their weight daily before training. She claims that she obeys his order because she likes the sport. Later on, she started to train daily and started to have a strict meal plan where she eats only a quarter of bread and salad once a day during the night after fasting throughout the day. By that time, her body weight had decreased subjectively, and she and her coach were comfortable with weight loss. After 5 years of training, her grandfather passed away, and she stopped exercising for 2 months. After that, she stopped going to the Taekwondo school because of an argument with the coach, but she continued to practice herself on a fixed daily schedule. She then joins the university and claims to practice sports there for the first 2 years. She stated that there were different occasions when she stopped exercising, but after a while, she worried about her weight gain and returned to exercise. Her mother states that “she always mentions that she wants to be thin and healthy; for this reason, she measures her weight very often. In addition, she gets worried a lot even if puts up a small amount of weight”. There was a time when she gained weight during her first‐year stay at university and when she returned to her hometown, she was worried a lot and ashamed of her body shape, so she avoided going outside and meeting her friends. She avoids wearing her previous regular outfits; rather, she wears a wide dress to cover her body throughout her stay. Subsequently, her weight decreased significantly to approximately 20 kg within the past 2 years and 10 kg within the past 6 months, and states to use semisolid foods during this period. She had amenorrhea for the past 2 years.

Her current body weight is 30 kg, her height is 151 cm and her BMI is 13.15 kg/m^2^, she states that her current body weight does not concern her. She claimed to have felt healthier than ever before. She also had a low mood that lasted for most of the days almost every day, loss of interest in pleasurable activities such as exercising and meeting with her friends, fragmentation of sleep, hopelessness, and recurrent suicidal thoughts, which persisted for a month. She claimed to have four different such episodes: The first one was while she was a grade 11 student after her grandfather, who was close to her passed away, stated that it was a difficult time for her. She reported that the other three episodes had occurred while she was a university student. Each episode lasts for 1–2 months, the main triggering factor for her episodes was not achieving things as she planned, and she claims to be a very scheduled, perfectionist who emphasizes a little detail for these, if things did not go as she planned she states to During the episodes, she feels low, worthless. She also had one suicide attempt by overdosing on medication (unspecified antibiotic) around 100 tabs after she planned and bought it from a pharmacy; after the attempt, she experienced nausea, vomiting, and fatigue and improved without any treatment. The attempt was during COVID time she was at home and she was feeling sad due to the frequent arguments with her mother, and she took the medication right after an argument. Her mother found her and took her to hospital. In the hospital only took Normal saline and did not receive any other treatment.

Six months later, she was a final‐year student at university, at which time she started to have a burning type of epigastric pain, loss of appetite, nausea, frequent vomiting, and significant weight loss. For these symptoms, she was taken to a local health center where baseline investigations, complete blood count (CBC), stool analysis, and Helicobacter pylori tests were performed. All were nonrevealing, and she was treated empirically with different medications (antiacids, proton pump inhibitors(PPIs)) at different times, which she did not respond to; she returned to Addis Ababa for possible better treatment options; her mother stated that on her first site, she even did not notice her child that she significantly lost weight, was lethargic and had easy fatigability while doing some minor activities at home, she later had even difficulty talking verbally, and she used gestures to communicate; for these, she was taken to a gastrointestinal specialty clinic where she was investigated with CBC, Liver function test (LFT), Renal function test (RFT), Abdominopelvic ultrasound, endoscopy, and fluoroscopy where all results were unremarkable. She was linked to the psychiatry clinic, based on her history, physical, mental state exam, and investigations, she was diagnosed with major depressive disorder + rumination disorder, and she was given, fluoxetine 20 mg po morning. She took the medication for 1 month and discontinued it later. She said she discontinued the medication because she had no response 2 months before the current presentation. Her symptoms worsened and had significant weight loss and loss of energy to the level where she spent the day and the night on her bed, and her mother brought her to the psychiatry outpatient department (OPD) without her will, where admission was offered and admitted after having a mutual discussion with the patient and her mother.

### 2.1 Laboratory investigation

Routine laboratory tests, including CBC, LFT, RFT, and electrolyte, were performed and were grossly normal. Close monitoring is shown in Table 1. Thyroid‐stimulating hormone (TSH) (1.29), total protein (8.69), albumin (4.9), random blood sugar (RBS) (82.5), and lipase (46.7) were all within the normal range. Abdominopelvic ultrasound and endoscopy were normal. The initial amylase level increased to 145, which subsequently decreased. The vitamin B12 was 148 which was low see the summary investigation (see Table 1).

**TABLE 1:** Summary of laboratory investigation.

Date	CBC		LFT		RFT		Electrolyte		Other	
14/11/2022	WBC	2.25X10^3^	AST	16.53	Urea	26.01	K+	3.79	Abdominopelvic U/S	Normal
	HGB	14.3 g/dL	ALT	9.8	Creatine	0.69	Na	138.2	Endoscopy	Normal
	HCT	38.85%	ALP	131			Cl	78.4		
	MCV	92 fl	Total Bilrubin	0.57			iCa	0.76		
	PLT	226 × 10^3^	Direct Bilrubin	0.12			tCa	1.87		
	TSH	1.29	Albumin	4.9						
			Total Protein	8.69						
			RBS	82.53						
28/11/2022							K+	5.4		
							Na	138		
							Cl	109.4		
							Mg	1.03		
							Amylase	145		
							Lipase	46.7		
							Direct Bilrubin	0.05		
14/12/2022	WBC	3.0 × 10^3^	AST	26.6	Urea	29	K+	4.58		
	HGB	14.2 g/dL	ALT	10.7	Creatine	0.55	Na	137		
	HCT	43%	ALP	55			Cl	99.2		
	MCV	92.6 fl								
	PLT	125 × 10^3^					Amylase	31.9		
							Lipase	41.1		
28/12/2022	WBC	2.36 × 10^3^							Vit B12	<148 (Normal = 187–883)
	HGB	13.2 g/dL					K+	4.75		
	HCT	39.9%					Na	144		
	MCV	96 fl					Cl	103		
	PLT	230 × 10^3^								

### 2.2 Physical and neuropsychological evaluation

During her psychiatric evaluation at admission, she was calm, cooperative, and well‐groomed with good eye contact, and psychomotor slowing was observed. The patient's speech decreased in tone, amount, rate, and volume. The patient had euthymic, mood‐congruent, and appropriate effects. The patient was coherent and had no delusions or suicidal thoughts. She was alert and oriented toward the place, person, and time. The fund of knowledge and abstract thinking was intact. She had fair judgment, but poor insight. Physical examination revealed that the general appearance was emaciated and chronic. Her weight, height, and body mass index were 30 kg, 151 cm, and BMI of 13.15 kg/m^2^. Baseline vital signs were BP 70/50, PR 52 beats per minute; RR, 14 breaths per minute; and T^o^, 36.1^o^c.

### 2.3 The course of the illness and management

With the diagnosis of extreme anorexia nervosa, restricting type; adult malnutrition; MDD (in remission); low risk of aggression; low risk of suicide; severe functional impairment she was admitted to the psychiatry ward with strict following‐up her vital signs, RBS, and weight subsequently. The general management principles implemented in this patient include; assessment of medical complications, weight restoration, psychological intervention, medication for comorbid depression, and long‐term psychological and biological treatment follow‐up to avoid relapse. After consultation with a nutritionist, nutritional rehabilitation management was started with frequent semisolid foods, and later to meet daily calorie needs, a high‐calorie nutritional supplement ensure was started with a daily meal plan. Nonstructured CBT and supportive therapy were administered weekly. Planned consideration of pharmacological options after stabilization. After stabilization, escitalopram (10 mg) was initiated with close monitoring of her vital signs and potential medication side effects.

## 3 DISCUSSION

Anorexia has been widely reported in industrialized and Western cultures when compared to developing countries.^2^, ^8^, ^11^ In the era of globalization, the availability of easy access to the Internet and international media has changed body image portrayal in developing countries like Ethiopia.^2^, ^8^ The previous notion that anorexia nervosa only occurs in the Western culture that idealizes thinness^8^ seems to contradict the case detection being seen in other developing countries as well as our settings. The case report presented a 23‐year‐old patient presented with a significantly low body weight based on a BMI of 13.15 kg/m^2^ and lost around 10 kg within the past 6 months which is a severely lower limit of normal body weight in adults. She also demonstrates an intense fear of gaining weight or becoming fat. For this, she has the presence of persistent behaviors like limiting her daily food intake, controlling portion size of food, only eating at certain times of the day or a certain amount of time after the last meal, and following other rigid rules that govern her eating behaviors and exercising excessively with achieving or lowering her weight. The low weight and the symptoms were present for more than 6 months. As illustrated by the case the patient has significant weight loss resulting in malnutrition as a result of restricting the food intake. After ruling out medical causes. The diagnosis of extreme anorexia nervosa, restricting type; adult malnutrition was given according to DSM5‐TR. Evidence suggests the age of onset for anorexia nervosa ranges between 12 and 20 years.^21^ The onset of symptoms of food restriction in this patient started 9 years back at her adolescent age(14 years of age) which is consistent with the literature. Furthermore, she was participating in taekwondo exercises, and her coach was strict on weight gain. A study suggests that eating disorders are high among adolescent and adult athletes, especially those involved with strict weight monitoring.^19^ Furthermore, a higher prevalence of eating disorders is seen among females athlete compared with males.^19^ A study suggested that the sports environment can make athletes vulnerable to risk factors of anorexia nervosa and other eating disorders. The study identifies sport‐specific risk factors like dieting and experienced pressure to lose weight, personality traits, early age at the start of training, impression motivation, and body weight preoccupied coaching style.^19^, ^20^

Our case is a trainee healthcare professional with a diagnosis of AN. A study done by Lebanese health science students and healthcare practitioners showed that the prevalence of eating disorders risk was 22.5% and about 80% of female students were high risk.^22^ The existing literature also supports the finding in the reported case.

In addition, she does not recognize the restriction of diet has severe medical risks associated with her current low body weight despite her physician and family repeatedly emphasizing the risks. She also has a vitamin B12 deficiency. Anorexia nervosa is a serious disorder that has the highest mortality compared to any psychiatric condition.^23^ Ego syntonic nature of the presentation most are unaware of the lethality of the state of weight loss. Anorexia nervosa affects every organ system and the medical complications include; cardiac complications like hypotension, peripheral edema, pericardial effusions, congestive cardiac failure, hypovolemia, and circulatory collapse.,^1^, ^23^ Electrolyte abnormalities include hypocalcemia, hyponatremia, hypokalaemia, hypomagnesemia, and hypophosphatemia; Anemia, Thrombocytopenia and rarely vitamin deficiencies pellagra (niacin deficiency) or scurvy (vitamin C deficiency)^1^, ^23^ etc.

The general management principles implemented in this patient include; the assessment of medical complications, weight restoration, psychological intervention, medication for comorbid depression, and long‐term psychological and biological treatment follow‐up to avoid relapse.

The first initial step that should be taken in anoxia nervosa is to establish and maintain a therapeutic alliance. The management teams should have a multidisciplinary approach involving psychiatrists, psychologists, internists, and nutritionists.^11^ Treatment of patients with anorexia nervosa should address the physical, psychological, and social aspects of the disorder.^23^ It is always important to rule out other medical causes of weight loss such as diabetes mellitus, endocrine diseases such as thyrotoxicosis and Addison's disease, and malignant conditions should all be excluded but should not delay the treatment of anorexia nervosa.^23^ Furthermore, stabilization of vital signs, electrolyte correction, nutritional rehabilitation, and motivation for recovery.^1^ Simple psychoeducation about the medical risk associated with anorexia nervosa helps with adherence to nutritional rehabilitation,^11^ and evidence suggests CBT reduces relapse risk for adults after weight restoration has been accomplished.^11^

Pharmacological treatment for anorexia nervosa has not shown any significant impact on weight gain but the use of antidepressants for those with mood symptoms has shown benefits. There are limited RCT studies on the pharmacological management of the adolescent population.^11^ The use of medication for acutely sick patients with anorexia nervosa should be avoided till stabilization.^23^

Therefore, we recommend a multidisciplinary approach involving psychiatrists, psychologists, internists, and nutritionists in the management of anorexia Nervosa. In addition, early screening and management of medical complications are crucial. Close monitoring of vital signs, restriction of caffeine, excess fluid, salt, and limiting excessive exercise. To assess for micronutrient deficiencies and vitamin supplements should be prescribed in the form of a multivitamin and thiamine preparation. The need for future training about positive coaching styles for coaches is mandatory to reduce the future impacts on young athletes. There is a need for future research on eating disorders to be undertaken in developing countries like Ethiopia.

## 4 CONCLUSION

The lifetime prevalence of anorexia nervosa ranges between 2.4 and 4.3 percent. In the era of globalization, the availability of easy access to the Internet and international media has changed body image portrayal in developing countries like Ethiopia. The presented case of a 23‐year‐old Ethiopian female patient who is athletic shows weight‐controlled sports activities and the manifestation of anorexia nervosa. Therefore, we recommend a multidisciplinary approach involving psychiatrists, psychologists, internists, and nutritionists in the management of anorexia Nervosa. In addition, early screening and management of medical complications are crucial. Close monitoring of vital signs, restriction of caffeine, excess fluid, salt, and limiting excessive exercise. Assessing for micronutrient deficiencies and vitamin supplements should be prescribed in the form of a multivitamin and thiamine preparation. The need for future training about positive coaching styles for coaches is mandatory to reduce the future impacts on young athletes. There is a need for future research on eating disorders to be undertaken in developing countries like Ethiopia.

## AUTHOR CONTRIBUTIONS

**Selamawit Alemayehu Tessema:** Conceptualization; data curation; methodology; supervision; writing – original draft; writing – review and editing. **Surafel Worku Megersa:** Conceptualization; methodology; writing – review and editing. **Meskerem Abebe:** Conceptualization; writing – review and editing. **Hailegiorgis Getnet:** Data curation; writing – review and editing. **Melat Tigabu:** Data curation; writing – review and editing.

## FUNDING INFORMATION

There was no available funding for the current study.

## CONFLICT OF INTEREST STATEMENT

The authors declare that there are no competing interests.

## ETHICS STATEMENT

Ethical clearance was obtained from the Department of Psychiatry at St. Pual's Hospital Millennium medical college. Ethical consent obtained from the patient is available with the authors upon request.

## CONSENT

Written informed consent was obtained from the patient for publication of this case report. A copy of the written consent will be available for review by the editor of this journal.

## Data Availability

“Not applicable”.
